# Effects of Bushen-Jiangya granules on blood pressure and pharmacogenomic evaluation in low-to-medium-risk hypertensive patients: study protocol for a randomized double-blind controlled trial

**DOI:** 10.1186/s13063-022-05999-2

**Published:** 2022-01-15

**Authors:** Xiaochen Yang, Lanping Liu, Xingjiang Xiong, Yun Zhang, Yongmei Liu, Hongzheng Li, Kuiwu Yao, Jie Wang

**Affiliations:** 1 Department of Health Care, Guang’anmen Hospital, China Academy of Chinese Medical Sciences, Beijing, China; 2 Department of Cardiology, Guang’anmen Hospital, China Academy of Chinese Medical Sciences, Beijing, China

## Abstract

**Introduction:**

Hypertension is one of the most important risk factors for cardiovascular disease, and its control rates remain low worldwide. The most effective strategy is that patients with hypertension should be diagnosed and treated early. Preliminary studies showed that the Bushen Jiangya granule (BSJY) could suppress ventricular hypertrophy and inflammatory responses, lower blood pressure, and protect the target organs of hypertension. We designed a randomized, double-blind, placebo-controlled trial to evaluate the efficacy of BSJY in patients with low-to-medium risk hypertension.

**Methods and analysis:**

This trial is a one-center, randomized, double-blind, placebo-controlled study. A total of 260 participants will be randomized in a 1:1 ratio to an experimental group (BSJY plus amlodipine) and a control group (placebo plus amlodipine). The trial cycle will last 8 weeks. The primary outcome is the change in 24-h average systolic and diastolic blood pressure. The secondary outcomes include heart rate variability, pharmacogenomic evaluation, improvement in TCM syndrome, and serum pro-inflammatory/anti-inflammatory cytokines between the two groups. The safety of medication will also be evaluated. All the data will be recorded in electronic case report forms and analyzed by SPSS V.22.0.

**Ethics and dissemination:**

This study has been approved by the Research Ethics Committee of Guang’anmen Hospital, China Academy of Chinese Medical Sciences in Beijing, China (No. 2019-186-KY-01). The participants are volunteers, understand the process of this trial, and sign an informed consent. The results of this study will be disseminated to the public through peer-reviewed journals and academic conferences.

**Discussion:**

We hypothesize that patients with low-to-medium-risk hypertension will benefit from BSJY. If successful, this study will provide evidence-based recommendations for clinicians.

**Trial registration:**

Chinese Clinical Trial Registry ChiMCTR1900002876. Registered in November 2019

**Supplementary Information:**

The online version contains supplementary material available at 10.1186/s13063-022-05999-2.

## Strengths and limitations of this study

- This is the first randomized, double-blinded, placebo-controlled clinical trial that explores the efficacy, safety, and pharmacogenomic evaluation of Chinese medicine (BSJY) in treating patients with low-to-medium-risk hypertension.
- Numerous experiments and case studies showed that BSJY had a good effect on lowering BP and improving quality of life. However, there is a lack of high-quality clinical research on the supposed benefits of BSJY.
- The complexity of the genetic mechanisms underlying hypertension and the need for much larger sample sizes when looking for genes associated with BP should be considered. For BSJY, it can be viewed as a very important environment element to pharmacogenomic evaluation.
- The multi-center experiments should be conducted in the future.

## Introduction

Hypertension is one of the most important public health issues due to its association with a number of serious diseases, including cardiovascular disease and stroke. According to the 2020 International Society of Hypertension (ISH) Global Hypertension Practice Guidelines, although effective management greatly reduces the risk of cardiovascular events, blood pressure (BP) remains uncontrolled in many people [1–3]. This ISH guide is different from the past, mainly in the following three points: (1) different BP goals: the best standard is that for young and middle-aged people, the target BP should be less than 130/80 mmHg (not less than 120/70 mmHg); (2) the grading of hypertension is simplified and reduced the three stages to the two main ones (stages 1 and 2); and (3) adjust the risk stratification, cancel the very high risk, and merge it into the high risk. BP remains uncontrolled in a number of people. If the diagnostic criteria for hypertension are moved forward, the BP of more people can be managed, which will help prevent long-term complications and reduce the long-term burden of economic health [4]. Although the new US guidelines advocate the priority lifestyle intervention for the hypertensive population with the new definition, there are big differences in implementation and treatment in China and other Asian counties, and large-scale clinical trials are still needed to determine the ideal antihypertensive treatment, not only for the senior people, but also for the young and middle-aged population [5].

For the treatment of hypertension, five drug classes (β-blockers, angiotensin-converting enzyme inhibitors, angiotensin receptor blockers, thiazide diuretics, and calcium channel blockers) are considered appropriate first-line therapy for HTN [6]. However, uncontrolled BP and resistance are observed in more than 40% of the patients, and more than 75% of patients require prescribing combination therapies and various medications [7, 8]. The low efficacy of some therapies could be related to inter-individual genetic variability. Novel targets for hypertension should focus on treating treatment-resistant hypertension, improving BP control, and targeting the genetic, functional, and structural alterations associated with hypertension [9]. Pharmacogenomic evaluation of antihypertensive responses offers the clinical promise of individualization of therapy based on a person’s genetic makeup [10, 11]. More than twenty single nucleotide polymorphisms (SNPs) have been associated with BP in genome-wide association studies in Asian people [12, 13]. The loci/SNPs associated with BP/hypertension are also associated with BP response to antihypertensive drugs [8].

Traditional Chinese medicine (TCM) has long been widely used in hypertension in China. *Huangdi Neijing*, a classic work on TCM, classified hypertension as headaches and vertigo. Nowadays, numerous studies have demonstrated the biological activity and therapeutic mechanism of TCM in hypertension [14, 15]. Bu-Shen-Jiang-Ya granule (BSJY) is composed of eight herbs, Dihuang (Radix Rehmanniae Glutinosae), Shanzhuyu (Corni Fructus), Duzhong (Cortex Eucommiae Ulmoidis), Tianma (Gastrodiae Rhizoma), Sanqi (Notoginseng Radix), Mudanpi (Cortex Radicis Moutan), Shanzha (Crataegi Fructus), and Zexie (Rhizoma Alismatis), which is made from a modification of the classical Chinese herbal formula Liuwei Dihuang pill (Table 1). Our previous studies show that BSJY has a clinical effect on patients suffering from kidney-yin deficiency syndrome type hypertension [38–40]. The experimental acute toxicology study showed that the safety of BSJY is reliable [41]. Besides, BSJY reversed hypertensive ventricular hypertrophy by regulating the ERK pathway and protected the endothelial function by regulating the PI3K/Akt pathway [42]. However, the pharmacogenomic evaluation of antihypertensive responses of BSJY remains unclear. This study is designed to investigate whether BSJY may represent a potential remedy for decreasing BP and slowing disease progression in low-to-medium-risk hypertension based on pharmacogenomic evaluation. If positive, this work will be the first one that provides an evidence-based medicine remedy for TCM on treating hypertension by pharmacogenomic evaluation.

**Table 1:** Components and dose of BSJY

Chinese name	English name	Latin name	Origin	Main ingredients	Main pharmacological effects	Raw drug weight (g)	Granule weight (g)
Duzhong	Eucommia bark	Cortex Eucommiae Ulmoidis	The bark of Cortex Eucommiae Ulmoidis	Quercetin; Mairin; beta-sitosterol; kaempferol; Erythraline; Eucommin A; (-)-Tabernemontanine; cyclopamine; GBGB; helenalin	Lowering BP [16]; reversing hypertensive vascular remodeling and hypertensive cardiac remodeling [17]; improving insulin resistance and lowering blood glucose [18]	10	0.48
Dihuang	Rehmannia	Radix Rehmanniae Glutinosae	The root of Radix Rehmanniae Glutinosae	EIC; sitosterol; stigmasterol; aeginetic acid; jioglutin D; rehmaglutin B	Lowering BP and improving insulin resistance [19] glucose metabolism, lipid metabolism [20]	25	3.57
Tianma	Gastrodia	Gastrodiae Rhizoma	The tuber of Gastrodiae Rhizoma	Daucosterol; citronellal; dauricine; gastrodin; p-hydroxybenzaldehyde; p-hydroxybenzyl alcohol; 4-hydroxybenzylamine; suchilactone; suffruticoside a; sucrose; vanillin; vanillin acetate	Impairing vascular endothelial function [21]; lowering BP [22]; improving lipid metabolism and insulin resistance [23]	20	1.82
Wuzhuyu	Cornus fruit	Corni Fructus	The fruit of Corni Fructus	Beta-sitosterol; sitosterol; stigmasterol; mandenol; ethyl linolenate; poriferast-5-en-3beta-ol; ethyl oleate (NF); leucanthoside; hydroxygenkwanin; telocinobufagin; gemin D; tetrahydroalstonine	Regulating adipogenesis [24]; lowering blood glucose and insulin resistance [25]; improving lipid metabolism [26]; protecting vascular endothelial cell [27] and protecting target organs and tissue related to diabetic damage [28]	10	1.43
Mudanpi	Cortex of the Peony Tree Rote	Cortex Radicis Moutan	The root and bark of Cortex Radicis Moutan	Quercetin; Mairin; sitosterol; kaempferol	Increasing the arterial blood flow, and improving glucose metabolism [29]; lowering BP and heart rate [30]	10	0.91
Zexie	Alisma	Rhizoma Alismatis	The rhizome of Rhizoma Alismatis	Sitosterol; alisol B; alisol B monoacetate; alisol,b,23-acetate; alisol B; alisol C; alisol C monoacetate; 1-monolinolein	Lowering blood glucose [31]; improving Hepatic lipid deposition [32]; improving lipid metabolism [33]	30	2.73
Sanqi	Notoginseng root	Notoginseng Radix	The root of Notoginseng Radix	Quercetin; beta-sitosterol; stigmasterol; mandenol; DFV; ginsenoside rh2; ginsenoside f2	Protecting the vascular endothelium [34]; improveing myocardial ischemia [35]	3	1.5
Shanzha	Crataegus fruit	Crataegi Fructus	The fruit of Crataegi	Quercetin; isorhamnetin; sitosterol; kaempferol; stigmasterol	Promoting anti-atherosclerosis [36]; improving vascular endothelial dysfunction [37]	30	4.29

## Methods and design

### Objectives

Our study aims to assess the clinical effect of BSJY on pharmacogenomics and pro-inflammatory/anti-inflammatory cytokines in patients with low-to-medium-risk hypertension, to firstly provide a preliminary pharmacogenomic evaluation of antihypertensive responses in hypertension, and to observe whether TCM plus Western medicine has a better curative effect than Western medicine alone.

### Study design

**Fig. 1:**
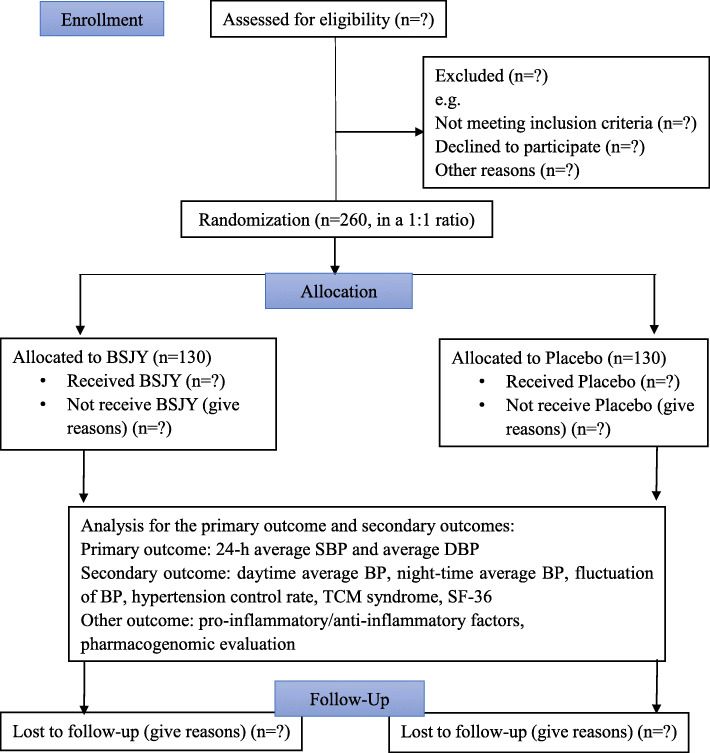
CONSORT flow diagram for BSJY clinical trial. BSJY, Bushen Jiangya granules

This protocol will be designed as a randomized, placebo-controlled trial. Participants, investigators, and statisticians will be blinded. A total of 260 subjects will be recruited at Guang’anmen Hospital of the China Academy of Chinese Medical Sciences in China. The trial will be implemented based on the principles of Good Clinical Practice and reported according to the CONSORT statement [43, 44]. The trial flow diagram is illustrated in Fig. 1. The Standard Protocol Items: Recommendations for Interventional Trials (SPIRIT) [45] Checklist is shown in Additional file 1. The SPIRIT-TCM Extension 2018 Checklist is shown in Additional file 3. This study has been registered at http://www.chictr.org.cn. (ChiMCTR1900002876).

### Patient and public involvement

This trial was designed to evaluate the effect of BSJY on BP and pharmacogenomic evaluation in low-to-medium-risk hypertension. Our previous clinical practice showed that BSJY adding to routine medications may lower BP and improve life quality in essential hypertension patients. The primary and secondary outcome measures used in this trial were considered as important endpoints in clinical practice. However, the participants in this trial were not directly involved in the design, recruitment, or conduct of the study. After the trial is completed, the results of this study will be disseminated to the public through peer-reviewed journals and academic conferences. The burden of intervention will not be assessed by trial participants.

### Participants

#### Inclusion criteria

The inclusion criteria include the following: stage I or II hypertension with low to medium risk, which is diagnosed according to the Chinese Hypertension Guidelines published in 2018 [46]; the BP was continuously or more than 3 times in a non-same day sitting position with systolic BP ≥ 140 mmHg and < 180 mmHg and (or) diastolic BP ≥ 90 mmHg and < 110 mmHg; TCM syndrome is associated with kidney deficiency syndrome (TCM kidney deficiency syndrome is shown in Additional file 2); male or female; without taking any hypotensive drugs; aged between 18 and 75 years; the participants are volunteers, understand the process of this trial, and sign an informed consent.

#### Exclusion criteria

The exclusion criteria include the following: uncooperative patient; secondary hypertension (symptomatic hypertension); severe hypertension systolic BP ≥ 180 mmHg and/or diastolic BP ≥ 110 mmHg; severe heart failure; insulin-dependent diabetes; infectious diseases; hyperthyroidism, combined with severe primary diseases including liver, kidney, hematopoietic system, nervous system, mental illness, and malignant tumors; patients participating in other clinical trials; pregnant or breastfeeding women; and with recent history of trauma.

#### Withdraw criteria

The following are the withdraw criteria:

1. Those who are unwilling to continue clinical trials during the research period
2. Those who did not follow the prescribed protocol during the study period
3. Those who have not completed the course of treatment or have incomplete information
4. During the study period, those who applied drugs other than those specified in the study plan that affect the efficacy of observation

### Ethics

The protocol (version 4.0, dated 09 October 2019) was approved by the clinical research ethics committee of Guang’anmen Hospital of the China Academy of Chinese Medical Sciences (approval 2019-186-KY-01), where the study will take place. It has been registered with the Chinese Clinical Trial Registry (ChiMCTR1900002876), which is listed in the WHO Registry Network. The Declaration of Helsinki and the principles of Good Clinical Practice were complied with by this trial [47, 48]. The participants must sign an informed consent form before enrollment. Meanwhile, they have the right to withdraw from the trial at any time.

### Randomization and blinding

The generated random tables were developed with Statistical Analysis System (SAS, version 9.4) by randomization biostatisticians. All the participants were included in the clinical trial according to the order of random table numbers. The ratio of the two groups was 1:1. For blinding, 260 sealed opaque envelopes were used to keep the allocation code for each participant, which were not accessible to the researchers, participants, clinical trial pharmacists, data managers, or statisticians. The BSJY granule was similar to its placebo in each group. The manufacturer, Sichuan Neo-Green Pharmaceutical Technology Development Co., Ltd., labeled the random codes on the package according to the principles of GCP.

### Intervention

BSJY (production batch number 191201), the placebo for BSJY (production batch number 191201), were produced and packed in a single batch by Sichuan Neo-Green Pharmaceutical Technology Development Co., Ltd. (Unified Social Credit Identifier 91510000684559613P). As tested, the drug conformed with the quality specified in the Chinese medicine standards published by the State Food and Drug Administration. Each bag of BSJY granule was 14.91 g and made from 138 g of the original drug. The main component of the placebo for BSJY is dextrin and 10% original drug. The BSJY and placebo granules (one bag at a time, two times per day, 8 weeks) will be provided by Sichuan Neo-Green Pharmaceutical Technology Development Co., Ltd. (Peng Zhou, China). Amlodipine besylate tablets (one tablet at a time, once per day, 8 weeks) were bought from Pfizer Co., Ltd. (Unified Social Credit Identifier 912102006048147187).

### Endpoint measurements

#### Primary outcome

The primary outcome is the change in 24-h average systolic and diastolic BP, 24-h coefficient of BP variability (CV), morning BP, 24-h BP trough/peak ratio (T/P), 24-h BP smoothing index (SI) [49].

#### Secondary outcomes

The following are the secondary outcomes:

1. Heart rates variability, based on routine 24-h BP monitoring
2. Assessment of any improvement in TCM Syndrome, using TCM Syndrome Integral Scale (Additional file 2). In this scale, the score of the symptom will be based on the severity, duration, and frequency of the symptoms: 0 points means the symptom did not appear in the past 1 month, 1 point means the symptoms are mild or the frequency of attacks is low, 2 points means the symptoms are moderate or the frequency of attacks is moderate; and 3 points means the symptoms are very severe or the symptoms constantly puzzled. The TCM therapeutic effect Index (TCMTEI), as calculated with the following formula, will be used to evaluate the treatment efficacy in the TCM syndrome.
  $$ \mathrm{TCMTEI}=\left(\frac{\mathrm{symptom}\ \mathrm{score}\ \mathrm{before}\ \mathrm{treatment}-\mathrm{symptom}\ \mathrm{score}\ \mathrm{after}\ \mathrm{treatment}\ }{\mathrm{symptom}\ \mathrm{score}\ \mathrm{before}\ \mathrm{treatment}}\right)\times 100\% $$
3. Assessment of any improvement in quality of life, using the 36-Item Short Form Health Survey [50, 51].
4. Changes in levels of blood lipids, including total cholesterol, triglycerides, low-density lipoproteins, and high-density lipoprotein.
5. Serum pro-inflammatory/anti-inflammatory cytokines in patients: changes in the serum levels of TNFα, IL2R, IL6, IL8, IL10, and IL1β will be assessed at baseline and treatment endpoint.
6. Pharmacogenomic evaluation: twenty loci/SNPs associated with BP/hypertension will be assessed at baseline and treatment endpoint.
7. Safety evaluation: routine blood and urine test including levels of creatinine, blood glucose, and homocysteine will be assessed at baseline and treatment endpoint.

### Data collection and management

The case report form (CRF) will be used for recording the process for each participant. In addition to the enrollment evaluation (− 7 ± 0 days), each participant will attend an evaluation visit when allocated and every 2 weeks afterwards during the trial (0 days, 2 weeks, 4 weeks, 6 weeks, and 8 weeks). The assessments including physical examination, improvement in symptoms, compliance with medications, questions about adverse events will be given to each participant. Blood tests and 24-h BP monitoring will be evaluated only at the enrollment and close-out visits. The schedule of enrollment, interventions, and assessments can be checked in Table 2. All researchers involved in data entry and data management will sign a confidentiality agreement to prevent data leakage. The personal information of all participants will be carefully protected, and the original CRF will be kept for 5 years after the end of the trial.

**Table 2:** Schedule of the data collection

Process/item	Run-in period	Run-in period	Pre-treatment	Treatment period			
Time point	− 7 day	− 1 day	0 days	2 weeks	4 weeks	6 weeks	8 weeks
**Baseline information**							
Informed consent	√						
Eligibility screen			√				
Medical history	√						
Allocation	√						
**Effectiveness observation**							
Blood pressure	√		√	√	√	√	√
24-h blood pressure			√				√
Pro-inflammatory/anti-inflammatory cytokines			√				√
Improvement in TCM syndrome			√	√	√	√	√
Improvement in quality of life			√				√
Pharmacogenomic evaluation			√				√
**Safety observation**							
Physical exam		√					√
Blood pressure		√					√
Routine blood test		√					√
Routine urine test		√					√
Liver and kidney function		√					√
Adverse event				√	√	√	√
**Other work**							
CRF audit							√

### Adverse events

Any accident, any signs of discomfort, or any disease symptoms, such as severe pain, syncope, hematoma, bleeding, or hypertensive emergencies, will be viewed as adverse events and will be recorded on the participant’s CRF. The research leader, sponsor, and the ethics committee will be contacted within 24 h, if the clinical researchers report an adverse event, and the ethics committee will recommend relevant treatment.

### Statistical analysis

#### Sample size calculation

The formula used to calculate the sample size is as follows, which is based on superiority clinical trial interval hypothesis test sample size estimation [52]. The sample size was calculated based on the expected reduction in reducing of BP. One previous study suggested that the reduction of BP after interventional treatment is 4.5 mmHg, and the BP SD is 10 mmHg. Therefore, we assume the reduction of BP as 4.5 mmHg in this study. In the following formula, *c* is the ratio between two sample cases, *n*1 = *n*2, so *c* = 1. *σ* is the BP SD 10 mmHg, and *δ* is the expected effect BP 4.5 mmHg, so *σ* = 10 and *δ* = 4.5. Given a type I error rate of *α* = 0.05, a power of 90% (type II error rate of *β* = 0.1), so *uα* = 1.96, *uβ* = 1.282. *n1* = *n2* ≈ 104, the sample size for one group needs to be 104, resulting in *n* = 2 × 104 = 208 patients. Considering the maximum possible dropout rate is 20%, a total of 260 patients needs to be allocated to reach the required number of patients for the efficacy analysis.

$$ {\displaystyle \begin{array}{c}{n}_1={\left[\frac{\left( u\alpha + u\beta \right)\sigma }{\delta}\right]}^2\frac{\left(1+c\right)}{c},{n}_2=c{n}_1\\ {}{n}_1={n}_2\approx 104\end{array}} $$

#### Planned data analysis

The intention-to-treat principle will be used to analyze the efficacy and safety of BSJY. The independent data administrator and the professional statistician will undertake data entry and data management, and perform the data analysis, respectively. All the efficacy and safety analyses with all randomly assigned participants included will be conducted within the full analysis set (FAS). The per-protocol set (PPS) analysis will also be conducted to compare the results from FAS and PPS. Demographic and laboratory characteristics will be calculated at baseline and after the treatment period for all patients. The statistical analysis will be done at Guang’anmen Hospital, China Academy of Chinese Medical Sciences in Beijing.

For continuous variables with normal distribution, the comparability of the characteristics between the two study groups will be assessed by using the *t*-test. While for the comparison of data with non-normal distribution, the non-parametric Mann-Whitney-Wilcoxon test will be used. Continuous variables will be expressed by mean ± SD. We will assess the paired *t*-test to compare the difference of the outcome between preintervention and postintervention in each group by using the independent *t*-test. A *χ*^2^ test will be used for categorical variables, while the Fisher exact test will be used when the theoretical frequency is less than 5 in more than 25% of the cells. Categorical variables will be shown as counts and percentages. Whether the hypothesis of superiority is available will be judged by comparing the 95%CI of the difference in intergroup efficacy. All statistical tests are unilateral tests; *p* < 0.05 is statistically significant. All statistical analyses will be performed using SPSS V.20.0.

## Discussion

Hypertension, as one of the most important risk factors for cardiovascular disease, and its treatment and control rates are still low worldwide [53]. Better treatment methods with fewer side effects are required, which was the motivation to apply BSJY in this research. In this trial, we will assess the efficacy and safety of BSJY in treating patients with low-to-medium risk hypertension.

According to our previous studies, BSJY can promote the expression of the ERK pathway and inhibit the expression of the TNF-α, MCP-1, and IL-6, which would suppress ventricular hypertrophy and inflammatory responses. Furthermore, BSJY may lower BP and protect the target organs of hypertension including the heart and kidney by inhibiting TGF-β1/Smads signaling molecules in spontaneously hypertensive rats [54]. Based on network pharmacology, 93 active ingredients were predicted for BSJY involving 566 core targets, including 50 direct targets, such as epinephrine receptor, adenosine receptor, endothelin receptor, nitric oxide synthase, and glucose kinases, involved in the regulation of calcium and sodium ion transport, vascular endothelial function, glucose and lipid metabolism, and other related biological processes and signaling pathways [55]. Liquid chromatography-mass spectrometry analysis of BSJY also has been conducted to evaluate its chemical components. It mainly contains tartaric acid, gallic acid, gastrodin, catalpol, neochlorogenic acid, 2-[4-(b-d-glucopyranosyloxy) benzyl] citrate, morroniside, chlorogenic acid, oxypaeoniflorin, cryptochlorogenic acid, loganin, parishin B, pinoresinol di-*O*-Glc, parishin C, quercetin-3-*O*-sambubioside, suffruticoside A or B or C or D, galloylpaeoniflorin, and iso-Mudanpioside H. However, there is a lack of high-quality clinical research on the supposed benefits of BSJY.

Pharmacogenomic evaluation of antihypertensive responses offers the clinical promise of individualization of therapy based on a person’s genetic makeup [11]. It is said that the loci/SNPs associated with BP/hypertension are also associated with BP response to antihypertensive drugs [10]. This is the first paper that researches pharmacogenomic evaluation of antihypertensive responses in TCM. The present study is designed as a double-blind, randomized, placebo-controlled trial that will provide high-powered evidence regarding the efficacy, safety, and pharmacogenomic evaluation of BSJY in treating patients with low-to-medium risk hypertension. The progress and quality of the trial will be monitored by a clinical research organization of Guang’anmen Hospital.

There are also some limitations to this study. First, we supposed to test twenty single nucleotide polymorphisms (SNPs) that have been associated with BP in genome-wide association studies in Asian people. It is different that significant genetic loci for BP and hypertension reported in genome-wide association studies in Europeans, Asians, and Africans. It should be considered that the complexity of the genetic mechanisms underlying hypertension and the need for much larger sample sizes when looking for genes associated with BP. For TCM, it can be viewed as a very important environment element to pharmacogenomic evaluation. Second, our multi-center experiments will be conducted in China in the future.

### Trial status

Patient recruitment began in November 2019 and was expected to be completed in December 2022. At the time of manuscript submission, 80 patients had been recruited and completed the 9-week follow-up. Currently, we are still recruiting participants. However, no analysis has been conducted since the commencement of the trial. No serious AEs have occurred to date.

## Supplementary Information

**Additional file 1:** SPIRIT Checklist.

**Additional file 2:** TCM kidney deficiency syndrome Scale.

**Additional file 3:** SPIRIT-TCM Extension 2018.

## References

[1] Unger T, Borghi C, Charchar F, Khan NA, Poulter NR, Prabhakaran D, Ramirez A, Schlaich M, Stergiou GS, Tomaszewski M, Wainford RD, Williams B, Schutte AE (2020). 2020 International Society of Hypertension Global Hypertension Practice Guidelines. Hypertension.

[2] Wang C, Yuan Y, Zheng M (2020). Association of age of onset of hypertension with cardiovascular diseases and mortality. J Am Coll.

[3] Chow CK, Gupta R (2019). Blood pressure control: a challenge to global health systems. Lancet.

[4] Wyss F, Coca A, Lopez-Jaramillo P, Ponte-Negretti C (2020). Task force for the management of arterial hypertension of the Interamerican Society of Cardiology (IASC); Reviewers from European Society of Hypertension (ESH), Latin-American Society of Hypertension (LASH), Spanish Society of Cardiology (SSC). Position statement of the Interamerican Society of Cardiology (IASC) on the current guidelines for the prevention, diagnosis and treatment of arterial hypertension 2017-2020. Int J Cardiol Hypertens.

[5] Luo D, Cheng Y, Zhang H, Ba M, Chen P, Li H, Chen K, Sha W, Zhang C, Chen H (2020). Association between high blood pressure and long term cardiovascular events in young adults: systematic review and meta-analysis. BMJ.

[6] Chobanian AV, Bakris GL, Black HR, Cushman WC, Green LA, Izzo JL (2003). National Heart, Lung, and Blood Institute Joint National Committee on Prevention, Detection, Evaluation, and Treatment of High Blood Pressure; National High Blood Pressure Education Program Coordinating Committee. The Seventh Report of the Joint National Committee on Prevention, Detection, Evaluation, and Treatment of High Blood Pressure: the JNC 7 report. JAMA.

[7] Tocci G, Presta V, Citoni B, Figliuzzi I, Bianchi F, Ferrucci A, Volpe M (2020). Blood pressure target achievement under monotherapy: a real-life appraisal. High Blood Press Cardiovasc Prev.

[8] Tocci G, Presta V, Ferri C, Redon J, Volpe M (2020). Blood pressure targets achievement according to 2018 ESC/ESH guidelines in three European excellence centers for hypertension. High Blood Press Cardiovasc Prev.

[9] Bhatt LK, Selokar I, Raut D, Hussain T (2021). Novel targets for hypertension drug discovery. Curr Hypertens Rep.

[10] Rysz J, Franczyk B, Rysz-Górzy ´nska M, Gluba-Brzózka A (2020). Pharmacogenomics of hypertension treatment. Int J Mol Sci.

[11] Gong Y, McDonough CW, Wang Z, Hou W, Cooper-DeHoff RM, Langaee TY, Beitelshees AL, Chapman AB, Gums JG, Bailey KR, Boerwinkle E, Turner ST, Johnson JA (2012). Hypertension susceptibility loci and blood pressure response to antihypertensives: results from the pharmacogenomic evaluation of antihypertensive responses study. Circ Cardiovasc Genet.

[12] Kato N, Takeuchi F, Tabara Y, Kelly TN, Go MJ, Sim X, Tay WT, Chen CH, Zhang Y, Yamamoto K, Katsuya T, Yokota M, Kim YJ, Ong RTH, Nabika T, Gu D, Chang LC, Kokubo Y, Huang W, Ohnaka K, Yamori Y, Nakashima E, Jaquish CE, Lee JY, Seielstad M, Isono M, Hixson JE, Chen YT, Miki T, Zhou X, Sugiyama T, Jeon JP, Liu JJ, Takayanagi R, Kim SS, Aung T, Sung YJ, Zhang X, Wong TY, Han BG, Kobayashi S, Ogihara T, Zhu D, Iwai N, Wu JY, Teo YY, Tai ES, Cho YS, He J (2011). Meta-analysis of genome-wide association studies identifies common variants associated with blood pressure variation in east Asians. Nat Genet.

[13] Lu X, Wang L, Lin X, Huang J, Charles Gu C, He M, Shen H, He J, Zhu J, Li H, Hixson JE, Wu T, Dai J, Lu L, Shen C, Chen S, He L, Mo Z, Hao Y, Mo X, Yang X, Li J, Cao J, Chen J, Fan Z, Li Y, Zhao L, Li H, Lu F, Yao C, Yu L, Xu L, Mu J, Wu X, Deng Y, Hu D, Zhang W, Ji X, Guo D, Guo Z, Zhou Z, Yang Z, Wang R, Yang J, Zhou X, Yan W, Sun N, Gao P, Gu D (2015). Genome-wide association study in Chinese identifies novel loci for blood pressure and hypertension. Hum Mol Genet.

[14] Wang J, Xiong X. Evidence-based Chinese medicine for hypertension, evidence-based complementary and alternative medicine. eCAM. 2013;(2013):978398. 10.1155/2013/978398 PMC3686073 23861720

[15] Xiong X, Yang X, Liu W, Chu F, Wang P, Wang J. Trends in the treatment of hypertension from the perspective of traditional Chinese medicine, evidence-based complementary alternative medicine. eCAM. 2013;275279.

[16] Luo L-f, Wu W-h, Zhou Y-j, Yan J, Yang G-p, Ouyang D (2010). Antihypertensive effect of Eucommia ulmoides Oliv, extracts in spontaneously hypertensive rats. J Ethnopharmacol.

[17] Yao Y, Wang Y, Zhang Y, Liu C (2017). Klotho ameliorates oxidized low-density lipoprotein (ox-LDL)-induced oxidative stress via regulating LOX-1 and PI3K/Akt/eNOS pathways. Lipids Health Dis.

[18] He K, Li X, Chen X, Ye X, Huang J, Jin Y, Li P, Deng Y, Jin Q, Shi Q, Shu H (2011). Evaluation of antidiabetic potential of selected traditional Chinese medicines in STZ-induced diabetic mice. J Ethnopharmacol.

[19] Dai X, Shulan S, Cai H, Wei D, Yan H, Zheng T, Zhu Z, Shang E-X, Guo S, Qian D, Duan J-A (2018). Protective effects of total glycoside from Rehmannia glutinosa leaves on diabetic nephropathy rats via regulating the metabolic profiling and modulating the TGF-β1 and wnt/β-Catenin signaling pathway. Front Pharmacol.

[20] Ren L, Xu Y, Qin G, Liu C, Wang S (2017). Effects of water extracts of Rehmannia glutinosa on antioxidant system of Nrf2 in paraquat-induced insulin resistance diabetic rat model. Exp Ther Med.

[21] Duan X, Wang W, Liu X, Yan H, Dai R, Lin Q (2015). Neuroprotective effect of ethyl acetate extract from gastrodia elata against transient focal cerebral ischemia in rats induced by middle cerebral artery occlusion. J Tradit Chin Med.

[22] Kho MC, Lee YJ, Cha JD, Choi KM, Kang DG, Ho SL (2014). Gastrodia elata ameliorates high-fructose diet-induced lipid metabolism and endothelial dysfunction, evidence-based complementary alternative medicine. eCAM.

[23] Liu Y, Gao J, Peng M, Meng H, Ma H, Cai P, Xu Y, Zhao Q, Si G (2018). A review on central nervous system effects of Gastrodin. Front Pharmacol.

[24] Kim H-L, Jeon Y-D, Park J, Rim H-K, Jeong M-Y, Lim H, Ko S-G, Jang H-J, Lee B-C, Lee K-T, Lee K-M, Lee H, Kim S-H, Kim S-J, Hong S-H, Um JY (2013). Corni fructus containing formulation attenuates weight gain in mice with diet-induced obesity and regulates adipogenesis through AMPK. Evid Complement Alternat Med.

[25] Wang D, Li C, Fan W, Yi T, Wei A, Ma Y (2019). Hypoglycemic and hypolipidemic effects of a polysaccharide from Fructus Corni in streptozotocin-induced diabetic rats. Int J Biol Macromol.

[26] Chen C-C, Hsu C-Y, Chen C-Y, Liu H-K (2008). Fructus Corni suppresses hepatic gluconeogenesis related gene transcription, enhances glucose responsiveness of pancreatic beta-cells, and prevents toxin induced beta-cell death. J. Ethnopharmacol.

[27] Gao D, Li Q, Gao Z, Wang L (2012). Antidiabetic effects of Corni Fructus extract in streptozotocin-induced diabetic rats. Yonsei Med J.

[28] Park CH, Noh JS, Park JC, Yokozawa T (2013). Beneficial effect of 7-O-galloyl-D-sedoheptulose, a polyphenol isolated from corni fructus, against diabetes-induced alterations in kidney and adipose tissue of type 2 diabetic db/db mice, Evidence-based complementary alternative medicine. eCAM.

[29] Do TH, Trung TN, Hien TT, Dao TT, Yim N, Ngoc TM, Keun OW, Bae KW (2010). Selected compounds derived from Moutan Cortex stimulated glucose uptake and glycogen synthesis via AMPK activation in human HepG2 cells. J Ethnopharmacol.

[30] Chen J, Hou X-F, Wang G, Zhong Q-X, Liu Y, Qiu H-H, Yang N, Jun-Fei G, Wang C-F, Zhang L, Song J, Huang L-Q, Jia XB, Zhang M-H, Liang F (2016). Terpene glycoside component from Moutan Cortex ameliorates diabetic nephropathy by regulating endoplasmic reticulum stress-related inflammatory responses. J Ethnopharmacol.

[31] Dan H, Wu J, Peng M, Hu X, Song C, Zhou Z, Yu S, Fang N (2011). Hypolipidemic effects of Alismatis rhizome on lipid profile in mice fed high-fat diet. Saudi Med J.

[32] Zhou X, Ren Q, Wang B, Fang G, Ling Y, Li X (2019). Alisol a 24-acetate isolated from the alismatis rhizoma improves hepatic lipid deposition in hyperlipidemic mice by ABCA1/ABCG1 pathway. J Nanosci Nanotechno.

[33] Ho C, Gao Y, Zheng D, Liu Y, Shan S, Fang B, Zhao Y, Song D, Zhang Y, Li Q (2019). Alisol A attenuates highfat-diet-induced obesity and metabolic disorders via the AMPK/ACC/SREBP-1c pathway. J Cell Mol Med.

[34] Chen YB, Dong YH (2006). Clinical effect and pharmacological analysis of panax notoginseng saponins in the treatment of hyperlipidmia. China Med Pharm.

[35] Xue-Jun DU, Lei Y, Yang J (2010). Effects of Radix ginseng and Radix notoginseng formula on expressions of vascular endothelial growth factor receptor-2 and hypoxia-inducible factor-1alpha in ischemic myocardium of rats with acute myocardial infarction. Zhong Xi Yi Jie He Xue Bao.

[36] Liu L-T, Zheng G-J, Zhang W-G, Guo G, Wu M (2014). Clinical study on treatment of carotid atherosclerosis with extraction of polygoni cuspidati rhizoma et Radix and crataegi fructus: a randomized controlled trial. Zhongguo Zhong Yao Za Zhi.

[37] Dalli E, Colomer E, Tormos MC, Cosín-Sales J, Milara J, Esteban E, Sáez G (2011). Crataegus laevigata decreases neutrophil elastase and has hypolipidemic effect: a randomized, double-blind, placebo-controlled trial. Phytomedicine.

[38] Yang XC, Xiong XJ, Wang J (2014). Clinical observation of 108 cases of primary hypertension treated with bu shen jiang ya therapy. World J Integr Traditional Western Med.

[39] Wang J, Xiong X (2012). Current situation and perspectives of clinical study in integrative medicine in china, Evidence-based complementary alternative medicine. eCAM.

[40] Wang J, Xiong X, Liu W (2014). Traditional chinese medicine syndromes for essential hypertension: a literature analysis of 13,272 patients, evidence-based complementary and alternative medicine. eCAM.

[41] Liu W, Wang J, Xiong XJ, Yang XC (2013). Experimental study of bu shen jiang ya decoction on acute toxicology. Beijing J Tridit Chin Med.

[42] Xiong X, Yang X, Duan L, Liu W, Zhang Y, Liu Y, Wang P, Li S, Li X (2017). Traditional Chinese medicine suppresses left ventricular hypertrophy by targeting extracellular signal-regulated kinases signaling pathway in spontaneously hypertensive rats. Sci Rep.

[43] Moher D, Hopewell S, Schulz KF, Montori V, Gøtzsche PC, Devereaux PJ, Elbourne D, Egger M, Altman DG, Consolidated Standards of Reporting Trials Group (2010). CONSORT 2010 explanation and elaboration: updated guidelines for reporting parallel group randomised trials. J Clin Epidemiol.

[44] Boutron I, Altman DG, Moher D, Schulz KF, Ravaud P, for the CONSORT NPT Group (2017). CONSORT Statement for Randomized Trials of Nonpharmacologic Treatments: a 2017 update and a CONSORT extension for nonpharmacologic trial abstracts. Ann Intern Med.

[45] Chan AW, Tetzlaff JM, Gøtzsche PC (2013). SPIRIT 2013 explanation and elaboration: guidance for protocols of clinical trials [J]. BMJ.

[46] Liu J (2020). Highlights of the 2018 Chinese hypertension guidelines. Clin Hypertens.

[47] World Medical Association Declaration of Helsinki (2001). Ethical principles for medical research involving human subjects. Bull World Health Organ.

[48] Switula D (2000). Principles of good clinical practice (GCP) in clinical research. Sci Eng Ethics.

[49] Zheng XY (2002). Guiding principles for clinical research of new Chinese medicines. China Med Sci Technol Press.

[50] Buysse DJ, Reynolds CF, Monk TH, Berman SR, Kupfer DJ (1989). The Pittsburgh Sleep Quality Index: a new instrument for psychiatric practice and research. Psychiatry Res.

[51] Insana SP, Hall M, Buysse DJ, Germain A (2013). Validation of the Pittsburgh Sleep Quality Index Addendum for posttraumatic stress disorder (PSQI-A) in U.S. male military veterans. J Trauma Stress.

[52] Wan X, Zh L, Liu JP (2017). Estimation of sample size in clinical studies: (1) clinical trials. J Tradit Chin Med.

[53] Ventura HO, Taler SJ, Strobeck JE (2005). Hypertension as a hemodynamic disease: the role of impedance cardiography in diagnostic, prognostic, and therapeutic decision making. Am J Hypertens.

[54] Liu W, Li Y, Xiong X, Chen Y, Qiao L, Wang J, Su X, Chu F, Liu H (2020). Traditional Chinese medicine protects against hypertensive kidney injury in Dahl salt-sensitive rats by targeting transforming growth factor-β signaling pathway. Biomed Pharmacother.

[55] Yang X-c, Yun Z, Yongmei L, Wang J (2021). Explore mechanism of Bushen Jiangya Decoction for hypertension based on network pharmacology. Chinses J Int Med Cardio-cerebrovasc Dis.

