# Supplementary material for: Effects of Bushen-Jiangya granules on blood pressure and pharmacogenomic evaluation in low-to-medium-risk hypertensive patients: study protocol for a randomized double-blind controlled trial
Source: Trials. 2022 Jan 15;23:37. doi: 10.1186/s13063-022-05999-2 (PMC8760657; doi:10.1186/s13063-022-05999-2)
Supplement: Supplementary file 2 — Additional file 2: TCM kidney deficiency syndrome Scale. [file 13063_2022_5999_MOESM2_ESM.pdf]

Additional file 2. **TCM kidney deficiency syndrome Scale**

**Follow the instructions in the table and mark with×in the☐of the conforming item**<br>**Treatment___week** **Evaluation time_____________**				
Symptoms or signs	No	Mild	Moderate	Severe
Dizziness	☐	Dizziness, Sporadic ☐	Rotation of sight, Unable to walk ☐	Dizzy, Unable to stand ☐
Headache	☐	Mild headache, Sporadic ☐	Headache is tolerable, Persistent non-remission ☐	Headache is unbearable ☐
Backache	☐	Backache in the morning, Activity can be reduced ☐	Continuous backache, Work harder ☐	Backache continues, Unable to relieve ☐
Knee weakness	☐	Occasionally knee weakness ☐	Unable to bear heavy objects ☐	Soft knees and unable to walk ☐
Fatigue and weakness	☐	occasionally slight fatigue ☐	Feeling weak ☐	Weakness, Unwilling to speak ☐
Hot hands, feet, and heart	☐	Hot hands, feet, and heart at night☐	Five upset fever ☐	Irritability, irritability, heat ☐
Palpitations	☐	occasionally slight palpitation ☐	Bursts ☐	Heart palpitations ☐
Insomnia	☐	Occasionally have many dreams or Time reduction ☐	Often insomnia ☐	unable to sleep ☐
Nocturia	☐	Nocturia 1 time ☐	Nocturia 2-3 times ☐	Nocturia more than 3 times ☐

Dry mouth	□	occasional attacks □	Dry mouth and less fluid □	I often feel dry mouth, Drink water all the time □
Forgetful	□	occasionally forget things, I can remember □	Often forget things, Not easy to remember □	Forget instantly, Can't remember □
Pale tongue	□	□	□	□
Moss white	□	□	□	□
Pulse count	□	□	□	□
Total score:				

Note: 0 points means: the symptom did not appear in the past 1 month; 1 point means: the symptoms are mild or the frequency of attacks is low; 2 points means: the symptoms are moderate or the frequency of attacks is moderate; 3 points: the symptoms are very severe, or the symptoms constantly puzzled.
