# Supplementary material for: Effects of Bushen-Jiangya granules on blood pressure and pharmacogenomic evaluation in low-to-medium-risk hypertensive patients: study protocol for a randomized double-blind controlled trial
Source: Trials. 2022 Jan 15;23:37. doi: 10.1186/s13063-022-05999-2 (PMC8760657; doi:10.1186/s13063-022-05999-2)
Supplement: Supplementary file 3 — Additional file 3: SPIRIT-TCM Extension 2018. [file 13063_2022_5999_MOESM3_ESM.pdf]

SPIRIT-TCM Extension 2018: Recommended Items for CHM Formulae as Experimental Interventions

Checklist	Item No	Description
**Fixed CHM Formulae**		
Yes	1	Name, source and dosage form (e.g., decoction, granules, powder, pills).
Yes	2	Name, source, processing method and dosage of each medical substance. Name of all substances should be presented in at least two types of languages: Chinese (Pinyin), Latin or English. Names of the parts of the substances used should be specified.
Yes	3	Authentication method of each ingredient, and how, when, where, by whom it will be conducted.
Yes	4	Production method of the formula.
Yes	5	Quality control of each ingredient and the whole formula.
Yes	6	Safety assessment of the formula, containing heavy metals and toxic elements test, pesticide residue test, microbial limit test, acute/chronic toxicity test.
Yes	7	Dosage of the formula, and how the dosage was determined.
Yes	8	Administration route (e.g., oral, external).
**Placebo Control**		
Yes	1	Name and dosage of each ingredient.
Yes	2	Description of the similarity of placebo with intervention (e.g., color, smell, taste, appearance, packing).
Yes	3	Quality control and safety surveillance, if any.
Yes	4	Administration route, dosage and regimen.
Yes	5	Production information: when, where, how and by whom the placebo will be produced.
